# Smartwatch Measures, Cognitive Function, and Older Adults: electronic Framingham Heart Study (eFHS)

**DOI:** 10.1093/geroni/igaf122.3653

**Published:** 2025-12-31

**Authors:** Jie He, Yuankai Zhang, Xuzhi Wang, Nicole Spartano, Jian Rong, Emelia Benjamin, Chunyu Liu, Joanne Murabito

**Affiliations:** Boston University School of Public Health, Boston, Massachusetts, United States; Boston University School of Public Health, Boston, Massachusetts, United States; Boston University School of Public Health, Boston, Massachusetts, United States; Boston University Chobanian & Avedisian School of Medicine, Boston, Massachusetts, United States; Boston University Chobanian & Avedisian School of Medicine, Boston, Massachusetts, United States; Boston University Chobanian & Avedisian School of Medicine, Boston, Massachusetts, United States; Boston University School of Public Health, Boston, Massachusetts, United States; Boston University Chobanian & Avedisian School of Medicine and Boston Medical Center, Boston, Massachusetts, United States

## Abstract

Smartwatches can continuously monitor heart rate (HR) and step counts. A recent report of wearable-derived daily heart rate per step (DHRPS) observed a stronger association with cardiovascular outcomes than either daily HR or steps alone. However, it remains unclear whether DHRPS is associated with cognitive function. We conducted a cross-sectional study with 249 older adults (mean age 73, cognitively intact, mean Mini-Mental State Examination score of 28.8; 56% women, 14% non-White; median follow-up 662 days [426, 818]) from eFHS with smartwatch data and neuropsychological (NP) assessments within 5 years of eFHS data collection (2021-2024). DHRPS (primary exposure; median 0.02) was calculated as daily average HR (median 74 [69, 78]) divided by daily step counts, averaged across all follow-up days. Secondary exposures included average daily “non-active” HR (median 68 [63, 73]) and average daily step counts (median 4810 [3270, 6831]). Cognitive function was assessed by a composite of NP tests (cNP) spanning memory, executive function, language, and visuospatial domains. Linear regression models were used to examine the association between exposures and cNP, adjusting for demographic, lifestyle, and health-related covariates. Each 1-SD higher DHRPS was associated with a 0.06-SD lower cNP (95% CI: -0.11 to -0.01; p = 0.011). Additionally, we found each 1000 more steps per day was associated with a 0.03-SD higher cognitive score (95% CI: 0.004 to 0.05; p = 0.023), but daily “non-active” HR was not (p = 0.77). Our findings suggest wearable-derived metrics like DHRPS may help identify early cognitive decline. Larger, longitudinal studies are needed to confirm our findings.

